# Structural and Functional Insights into CRF Peptides and Their Receptors

**DOI:** 10.3390/biology13020120

**Published:** 2024-02-13

**Authors:** Minos-Timotheos Matsoukas, Vasilis Panagiotopoulos, Vlasios Karageorgos, George P. Chrousos, Maria Venihaki, George Liapakis

**Affiliations:** 1 Department of Biomedical Engineering, School of Engineering, University of West Attica, 12243 Athens, Greece; vpanagiotop@uniwa.gr; 2 Department of Pharmacology, Faculty of Medicine, University of Crete, 71003 Heraklion, Greece; bkarageorgos@hotmail.com; 3 University Research Institute of Maternal and Child Health and Precision Medicine and UNESCO, National and Kapodistrian University of Athens, Livadias 8, 11527 Athens, Greece; chrousos@gmail.com; 4 Department of Clinical Chemistry, Faculty of Medicine, University of Crete, 71003 Heraklion, Greece; venycham@uoc.gr

**Keywords:** CRF-peptides, CRF-receptors, structure, activation, binding, agonists, antagonists

## Abstract

**Simple Summary:**

Corticotropin-releasing factor or hormone (CRF or CRH) belongs to the family of CRF peptide and non-peptide analogs (or CRF ligands), which play important roles in many physiologic and pathophysiologic conditions. Several of the CRF ligands have shown considerable therapeutic potential in the treatment of various diseases. The CRF ligands act by interacting with two types of receptors. This work describes the structure of CRF ligands and their receptors, as well as the mode of CRF ligand binding to receptors and the activation mechanism of the latter. Understanding the structural basis of CRF ligand binding and activation of their receptors opens avenues for the development of novel drugs targeting CRF receptors.

**Abstract:**

Corticotropin-releasing factor or hormone (CRF or CRH) and the urocortins regulate a plethora of physiological functions and are involved in many pathophysiological processes. CRF and urocortins belong to the family of CRF peptides (CRF family), which includes sauvagine, urotensin, and many synthetic peptide and non-peptide CRF analogs. Several of the CRF analogs have shown considerable therapeutic potential in the treatment of various diseases. The CRF peptide family act by interacting with two types of plasma membrane proteins, type 1 (CRF_1_R) and type 2 (CRF_2_R), which belong to subfamily B1 of the family B G-protein-coupled receptors (GPCRs). This work describes the structure of CRF peptides and their receptors and the activation mechanism of the latter, which is compared with that of other GPCRs. It also discusses recent structural information that rationalizes the selective binding of various ligands to the two CRF receptor types and the activation of receptors by different agonists.

## 1. Introduction

The human corticotropin-releasing factor or hormone (CRF or CRH), also known as h/r CRF, because the human sequence (hCRF) is identical to that of its rat counterpart (rCRF), is a peptide consisting of 41 amino acids. h/rCRF (or in general CRF) belongs to a family of peptides (CRF peptide family) from several species, such as mammals, amphibians, and fish, which includes ovine CRF (oCRF), Sauvagine (SVG), Urotensin (UI), Urocortin I (UcnI), Urocortin II (UcnII) and Urocortin III (UcnIII) [1,2,3,4,5,6,7]. The peptides of the CRF family act by interacting with two types of G-protein-coupled CRF receptors (GPCRs), type 1 (CRF_1_R) and type 2 (CRF_2_R), and their action is modulated by the CRF-binding protein (CRF-BP), which binds and inactivates them [1,8,9]. 

The peptide is secreted by the hypothalamus and it is transported to the pituitary, where it is responsible for the release of adrenocorticotropic hormone (ACTH) [10,11]. Subsequently, ACTH stimulates the release of glucocorticoids from the adrenals [10,11]. Hypothalamic CRF is essential for homeostatic maintenance by regulating the function of the hypothalamic–pituitary–adrenal (HPA) axis and orchestrating various responses to stress, including autonomic, neuroendocrine, immunologic, and behavioral ones [10,11]. Moreover, CRF plays a role in stress and other physiological processes by regulating the cardiovascular, gastrointestinal, reproductive, and central nervous (CNS) systems [12,13,14,15,16,17,18,19,20,21,22,23,24,25,26,27,28,29,30,31,32,33,34,35,36,37].

CRF is implicated in the pathophysiology of many psychiatric disorders, including depression, anxiety, post-traumatic stress disorder (PTSD), and substance/alcohol abuse [34]. The effects of CRF on anxiety and depression are predominately mediated through its interaction with CRF_1_R [15,38,39,40]. Several non-peptide CRF_1_R-selective analogs have been used to treat psychiatric diseases in preclinical studies and clinical trials [34,41,42,43]. In general, CRF_2_R acts in a manner that counteracts the effects of CRF_1_R, having anxiolytic-like effects following administration of the CRF_2_R-selective UcnII or UcnIII in experimental animals [44,45,46]. However, activation of CRF_2_R could differentially affect depression and anxiety, depending on the region of the CNS [33]. In addition to their CNS effects, preclinical studies have shown that CRF_1_R-selective antagonists could be possibly effective in the treatment of abdominal and pelvic diseases, whereas previous studies have shown that UcnI has cardioprotective properties and UcnII and UcnIII are potent vasodilators [36,37,47,48,49,50]. Moreover, the CRF_1_R-selective antagonists, antalarmin and tildacerfont (or LY2371712), have been shown to decrease the progression of endometriosis and the excessive androgen production in congenital adrenal hyperplasia (allowing for glucocorticoid dose reduction), respectively [51,52]. 

**Figure 1:**
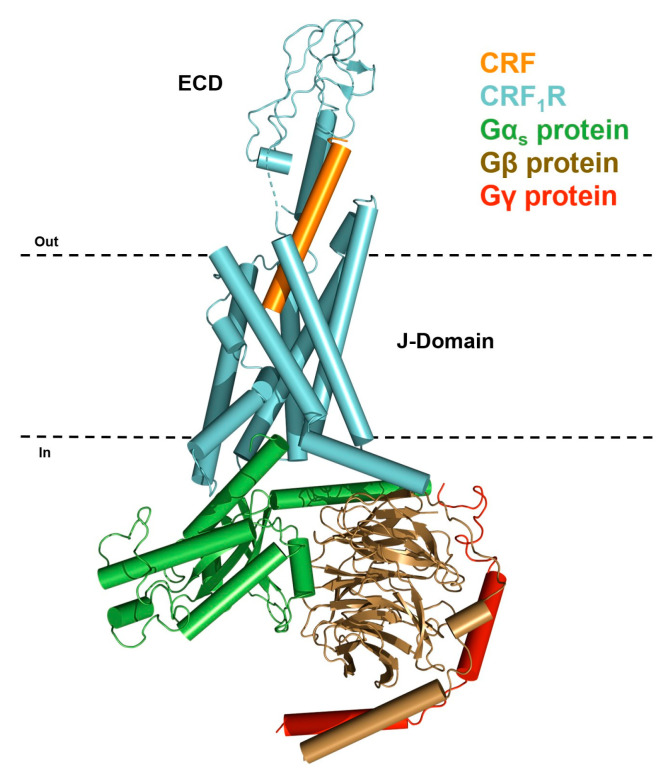
Model of CRF_1_R receptor (blue) in complex with the CRF (orange) and the three subunits of G-proteins, namely the G_αs_ protein (green), G_β_ protein (brown), and G_γ_ protein (red). This generalized model of receptor has been created based on the crystal structure of the J-Domain of CRF_1_R in complex with CRF peptide and G-proteins (PDB: 6P9X) and the crystal structure of the ECD domain of CRF_1_R in complex with the CRF (PDB: 3EHU). The place between the two dotted lines is the lipid bilayer of the plasma membrane.

The CRF receptors belong to the subfamily B1 of family B GPCRs [53]. Like all GPCRs, CRF receptors are plasma membrane proteins that contain seven alpha-helical transmembrane domains (TMs), and three extracellular (ELs) and three intracellular loops (ILs), as well as an extracellular amino-terminal domain (ECD) and an intracellular carboxyl–terminal region (C-domain) (Figure 1) [1].

The ECD is also named the N-domain, whereas their ELs along with their TMs comprise the J-domain (Figure 1) [1]. The TMs of CRF receptors are arranged such as to form a pocket into the phospholipid bilayer of the plasma membrane, named the binding pocket, the surface of which is accessible to the extracellular fluid and interacts with ligands [54]. The binding of CRF agonists to the extracellular domains of CRF receptors triggers conformational changes, which are associated with receptor activation and the subsequent stimulation of G-proteins, thus leading to a biological response (Figure 1). Importantly, activation of each type of CRF receptor could result in a diverse spectrum of biological responses, given that it could stimulate different signaling pathways by interacting with diverse G-proteins, such as G_αs_, G_αi_, G_αo_, G_αq_, and G_αz_ [55,56].

Even though CRF receptors and the other subfamily B1 GPCRs have a low sequence homology with family A GPCRs, all share a common structural architecture and several common activation mechanisms, suggesting that these characteristics have been conserved over the course of evolution, being very important for their function [53,57,58].

## 2. Pharmacological Properties of CRF Ligands and Their Receptors and Historical Overview

Pharmacological studies have shown that different CRF peptides display different binding affinities for the CRF_1_R and CRF_2_R (Table 1). The oCRF and all known non-peptide CRF analogs, including antalarmin (K_i_ = 9.7 nM, where K_i_ is the binding affinity of the ligand determined from heterologous displacement radioligand experiments [59]), bind selectively to CRF_1_R, whereas UcnII and UcnIII are CRF_2_R-selective ligands. Human UcnI (hUcnI) and SVG bind non-selectively to CRF_1_R and CRF_2_R. In addition, the synthetic CRF peptides, astressin, and α-helical CRF(9-41) (see below) interact with CRF_1_R and CRF_2_R with slightly different binding affinities [1]. 

The oCRF was first characterized and synthesized by Vale and his research team at Salk Institute, in 1981, whereas concurrently a similar peptide, named sauvagine, was isolated from the skin of Phyllomedusa sauvagei [3,10]. A year later, urotensin I, a CRF-like peptide with hypotensive properties, was isolated in Catostomus species [4]. Two years after the initial discovery of CRF, its rat counterpart was isolated by the same research team at Salk Institute, whereas concurrently a Japanese research group identified the gene encoding the human CRF [62,63]. Subsequently, a CRF-like peptide has been also identified and isolated from other species such as pig, fish, and frog [64,65,66]. Later on, and specifically in 1995, Vaughan et al. identified the rat UcnI, followed by the identification of its human counterpart and the homologous peptides UcnII and UcnIII from various species [2,5,6,7,67]. In addition to CRF peptides from different species, the CRF family includes synthetic peptides, such as stressin1-A, astressin, astressin-2B, antiSVG-30, and cortagine, and synthetic non-peptide CRF analogs, such as antalarmin. These CRF synthetic peptides are CRF_1_R-selective, CRF_2_R-selective, or non-selective agonists or antagonists and were derived from appropriate modifications of the maternal peptides, as reviewed by Liapakis et al. 2011 [1]. In addition to CRF peptide analogs, non-peptide CRF_1_R-selective antagonists were created as discussed below. 

## 3. Structural Features of CRF Family Peptides

### 3.1. Peptide CRF Analogs

**Figure 2:**
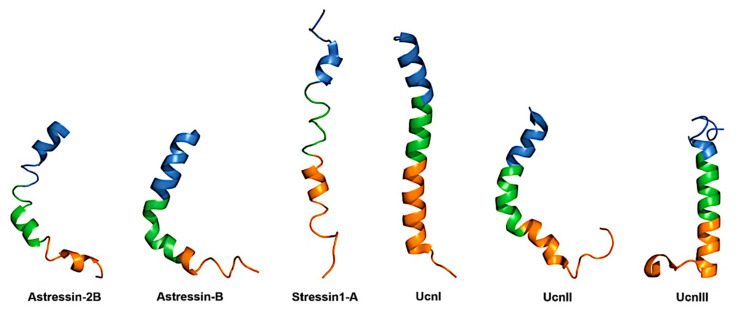
Structures of CRF analog peptides Astressin-2B (PDB: 2RM9), Astressin-B (PDB: 2RMD), Stressin1-A (PDB: 2RME), UcnI (PDB: 2RMF), UcnII (PDB: 2RMG) and UcnIII (PDB: 2RMH). The C-segment of the peptides is depicted in blue color, whereas the I-segment is depicted in green color and the N-segment is depicted in orange color [68].

The peptides of the CRF family consist of three segments: a carboxy-terminal (C-segment), an amino-terminal (N-segment), and an internal (I-segment) one. A study from Grace et al. (2007) describes the NMR structures of six CRF peptide analogs (the antagonists astressin-2B and astressin-B, the agonist stressin1-A, and the natural ligands human UcnI, UcnII, and UcnIII) in solution (Figure 2) [68].

#### 3.1.1. The N-Segment

The N-segment of CRF peptides is significant for their biological activity. Removal of the amino-terminal 23 or 28 amino acids from UI created biologically inactive analogs [69]. Similarly, deletion of the eight amino-terminal amino acids of oCRF resulted in the inactive peptide oCRF (9–41) [70]. This was likely due to the loss of the ability of the truncated peptide to produce a biological effect and not to its binding to CRF receptor, granted that it was able to antagonize the biological actions of CRF [70]. Further modifications of the truncated oCRF (9–41), which enhanced its α-helical structure, created the first CRF antagonist, named α-helical-CRF (9–41) [70]. Supportive evidence for the functional importance of the N-segment of CRF peptides has been provided by the study of Kornreich et al., which has shown that Ala mutation of most amino acids in this region had detrimental results on the biological activity of CRF [71]. 

#### 3.1.2. The C-Segment

The C-segment is also very important for the function of CRF peptides and their binding to receptors. The CRF analog, YAM19, which consists of only the 12 C-terminal amino acids of CRF, bound with high affinity to a soluble form of the ECD of CRF_1_R [72]. In marked contrast, deletion of the two last amino acids of its C-segment, Ile40, and Ala41, largely decreased the biological potency of CRF [10,71].

Similarly, deletion of the amino acid at position 41 from a CRF-truncated antagonist decreased its antagonistic activity [73]. Furthermore, the removal of the five carboxyl-terminal amino acids (37–41) from UI or substitution of a free acid for the amidated carboxyl-terminal end of CRF reduced (or abolished) the biological activity of the peptides [10,69,71,74].

Supportive evidence for the functional importance of the C-segment of CRF peptides is provided by several studies. Truncation of the carboxyl-terminal segment of UI, resulting in a smaller fragment, containing residues 1–19, largely decreased the activity of the peptide [69]. Similar to UI, the stepwise deletion of C-terminal amino acids from astressin, thus resulting in different fragments having detrimental effects in the peptide binding [73]. Importantly, even the deletion of only the last C-terminal residue (Ile41^P^) largely reduced the binding affinity of the peptide [73]. In addition, Ala substitutions for Arg35^P^ and Leu38^P^ in the C-segment of CRF significantly reduced the potency and binding affinity of the peptide [10,71,74]. The superscripts of peptide residues represent their position in the peptide sequence followed by the letter p, which is the abbreviation of the peptide. For example, Arg^35P^ indicates that Arg is the 35th amino acid in the peptide sequence, starting from the N-terminus. Substitution with alanine is used to determine the role of side chains because it removes all side-chain atoms past the β-carbon [75]. Consistent with the functional importance of residue 38, its replacement in peptide-1, a small 12-amino-acid N-truncated analog of CRF, by cyclohexylalanine (Cha) increased its affinity for CRF_1_R [76]. In contrast, the substitution of residue 38 by the smaller Phe did not increase its affinity, suggesting that the bulkier Cha maximized the hydrophobic interaction between residue 38 of the peptide and the receptor [76]. It is possible that in small peptides with fewer interactions with the CRF_1_R, the side chain of residue 38 plays a crucial role in this network of interactions.

The significant role of Arg^35^^P^ in its interaction with the receptor is supported by the crystal structure of the CRF_1_R in complex with CRF, in which a network of interactions take place between Arg^35P^ of CRF and receptor residues, as thoroughly discussed below [74].

#### 3.1.3. The I-Segment

**Figure 3:**
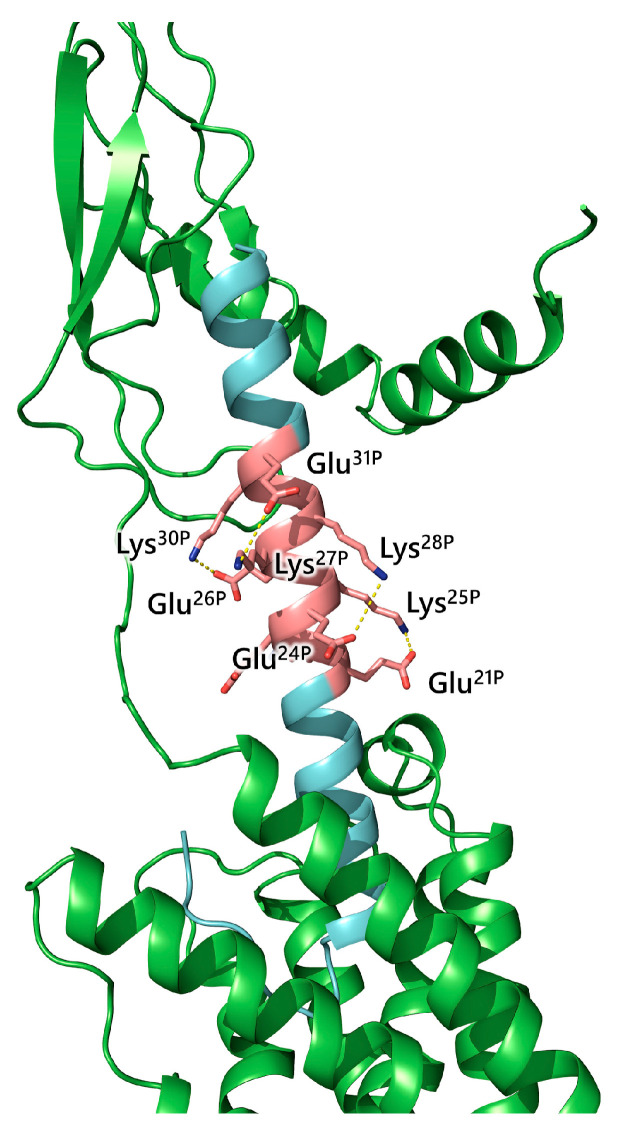
Model of CRF_1_R receptor (green) in complex with UcnI-EK (cyan). Salt bridges between peptide amino acids are shown in pink sticks.

An important role for the CRF_1_R binding likely plays the I-segment of peptides containing amino acids 14-30. Beyermann et al. created the analog UcnI-EK (UEK) by replacing the I-segment of UcnI with a highly charged linker consisting exclusively of Glu acid and Lys, which were arranged in a way that side chains at positions i and i4 form salt bridges (EKEEKEKKRKE) (Figure 3) [77]. This arrangement resulted in helix stabilization. Stepwise shortening of this charged linker by deleting amino acids resulted in peptide analogs of various lengths, with or without a complete alpha-helical conformation (Table 2). Only peptides bearing a complete a-helical linker, independently of its length, are potent, suggesting that the orientation of the two N- and C-segments rather than the preservation of a specific distance between them is important for peptide function [77].

Supportive evidence for the functional significance of the I-segment of CRF peptides has been provided by previous studies, which have shown that a segment between residues 8 and 32 could form an α-helical structure, which is likely stabilized in less hydrophilic environments, such as the amphiphilic one of cell membrane containing the CRF binding sites [78,79]. In addition, enhancement of the α-helical structure of peptide after the substitution of α-helical preferring residues with several amino acids of oCRF, increased the biological potency of the peptide [70]. In contrast, replacing several amino acids of oCRF with their D-enantiomers drastically decreased its biopotency [80,81]. D-enantiomers destabilize the α-helical structures [82]. The α-helical structure of CRF peptides has been verified in an NMR study (Figure 2) [68].

The importance of the I-segment of CRF peptides is further supported by the study of Eckart et al. (2001) [83]. Specifically, the Ala^22P^ of the Ala^22P^-Arg^23P^-Ala^24P^-Glu^25P^ (ARAE) motif of the α-helical I-segment of CRF is very important for peptide binding to the CRF-BP. Mutation of the Ala^22P^ to the corresponding Glu of SVG changed the phenotype of CRF to that of SVG, by largely reducing the binding affinity of peptide to CRF-BP. 

### 3.2. Non-Peptide CRF Analogs 

**Figure 4:**
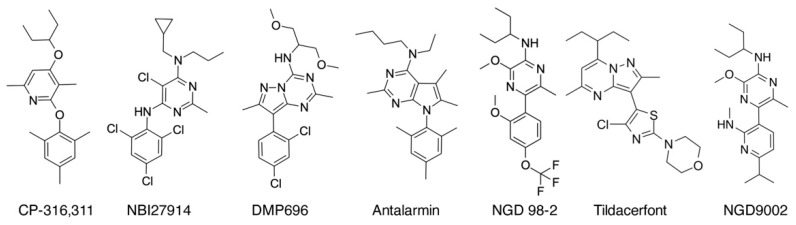
Structures of reported small molecule antagonists of CRF_1_R [84,85,86,87].

In addition to peptide ligands, many non-peptide antagonists have been created. Most of these analogs have a planar heterocyclic structure (mono-, bi- or tri-) in the center of the molecule [84,85,86,87] (Figure 4). Their heterocyclic ring contains a nitrogen atom, which could participate in hydrogen bonds and it is an important functional element of these analogs. In addition to this nitrogen atom, functionally important groups are a lipophilic one attached at the top of the core, and a lower aryl or heteroaryl ring (Figure 4). Another principal functional element is an ortho-substituent in the lower ring, which restricts this ring orthogonal to the plane of the core (Figure 4). This restriction could also be accomplished by adding an alkyl or alkoxy group next to its nitrogen atom into the core. Moreover, new compounds have been synthesized, bearing an acyclic central core with a nitrogen atom [88]. The important role of this nitrogen atom was suggested by its methylation abolished binding [88]. The latest CRF_1_R small-molecule antagonists reported are NGD9002 and NGD98-2 (Figure 4), which suppressed stimulation of motor activity of the colon induced by stress and visceral hypersensitivity in experimental animals [87]. However, small allosteric molecules have failed to reach clinical utility due to limitations arising from their similar chemical properties [89].

## 4. Receptors

### 4.1. The ECD

**Figure 5:**
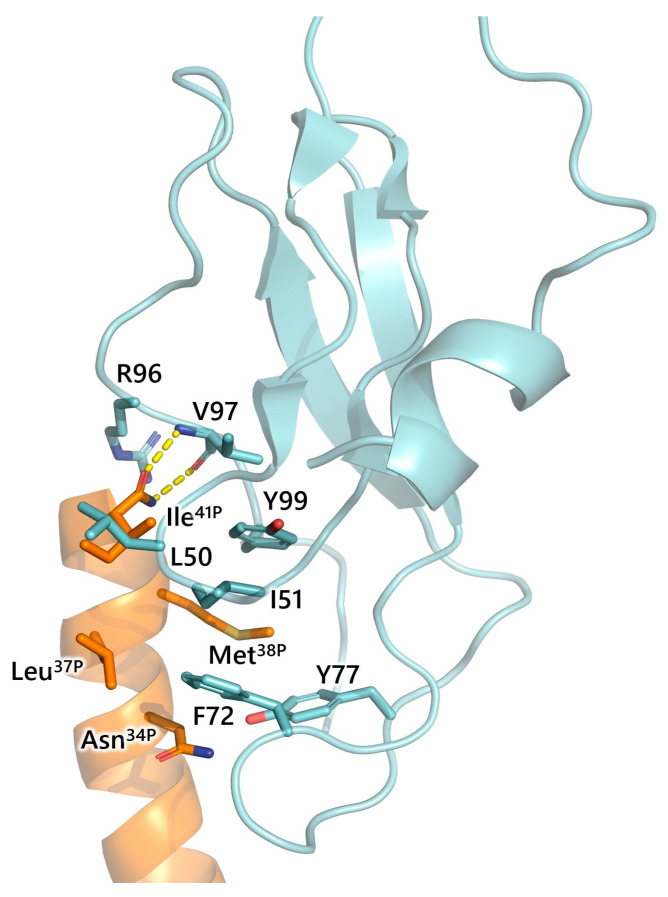
Crystal structure of the ECD (cyan) of human CRF_1_R (PDB: 3EHU) in complex with CRF (orange). Among the interactions between ECD and peptide, functionally important ones are the H-bonds (yellow sticks) between the main chain atoms of Val97, and the oxygen and nitrogen atoms of the C-terminal amide group (of residue 41) of CRF.

The extracellular amino-terminal domain (or ECD) of CRF_1_R plays a major role in ligand binding. Replacement of the ECD of CRF_1_R with the ECD of the receptor for the GH-releasing hormone (GRF-R) resulted in the loss of astressin and UcnI binding [90]. In marked contrast, replacement of ECD of the GRF-R with the ECD of CRF_1_R generated a receptor capable to bind astressin and UcnI [90]. Supportive evidence for the significance of ECD in CRF peptide binding is provided by previous studies, which have shown that a soluble form of the isolated ECD of CRF_1_R and CRF_2_R, and a receptor formed by substituting the ECD of the receptor for activin receptor (a single transmembrane protein) with the corresponding region of CRF_1_R or CRF_2_R, bind various CRF peptides with considerable affinities [91,92,93]. A crystal structure of the isolated ECD of CRF_1_R revealed that main chain atoms of Val97 form hydrogen bonds with the oxygen and nitrogen atoms of the C-terminal amide group (of residue 41) of CRF, demonstrating its important role in peptide function (Figure 5) [74]. In addition, Ile^41P^ of CRF hydrophobically interacts with residues Leu50 and Ile51 of the ECD. Furthermore, hydrophobic interactions between Met^38P^ of CRF and Tyr99 and Tyr77 of the ECD have been observed, as well as hydrophobic interactions along Phe72 of the ECD with Leu^37P^ of CRF. The Asn^34P^ of CRF could also possibly form a hydrogen bond with the side chain oxygen of Tyr77 of the ECD. Moreover, the nitrogen of the C-terminal amide interacts through a hydrogen bond with the backbone carbonyl of Met38 of CRF, which stabilizes the significant α-helical structure of the peptide [74] (Figure 5). Similarly, the amide group of the C-terminal residue 41 of astressin forms H-bonds with CRF_2_R residues including Val113 (Val97 in CRF_1_R) [94].

In addition to these amino acids, several Cys of the ECD of CRF_1_R and CRF_2_R form disulfide bridges that play an important functional role [74,92,93,95,96]. A reduction in these disulfide bonds in CRF_1_R using DTTs or their mutation decreased CRF binding [97]. These disulfide bonds hold important structural motifs in the ECD of CRF_1_R. These structural motifs are a short N-terminal α-helix followed by two anti-parallel β-sheets each with two β-strands (β1-β2 and β3-β4), and a short C-terminal α-helix, as revealed in the crystal structure of the ECD of CRF_1_R (Figure 5) [74]. A similar structure has been observed in the structure of the ECD of CRF_2_R, as determined in an NMR study, with some differences, such as the absence of a salt bridge between Arg85 and Asp49 in CRF_1_R, as observed in CRF_2_R [74,94]. 

These structural motifs of the receptor’s ECD are of vital importance for its function. Specifically, the β1-β2 loop of CRF_1_R, which is formed between the β1-β2 strands and interacts with the Met^38P^ and Ile^41P^ of CRF, is shifted closer to CRF upon peptide binding, rendering the Ile51 at the top of this loop a contact site of the peptide [74]. Structural rearrangements are also associated with peptide binding to the CRF_2_R [92,94].

In addition to its significance in ligand binding, the ECD is proposed to play a role in CRF receptor activation by interacting with the third extracellular loop (EL3) of the J-domain of receptor [98]. Specifically, Dore et al. proposed that the EL3 of CRF_1_R likely interacts with the ECD to stabilize an inactive conformation of the receptor, like the glucagon receptor [98,99]. Upon CRF binding to the ECD, the peptide destabilizes the receptor’s inactive state by affecting ECD-EL3 interaction before interacting with the J-domain to result in receptor activation. In accordance with this theory, a recent cryo-EM study of the active states of both CRF_1_R and CRF_2_R in complex with UcnI has shown that the ECDs do not interact with the TMs of receptors [100].

### 4.2. The J-Domain

**Figure 6:**
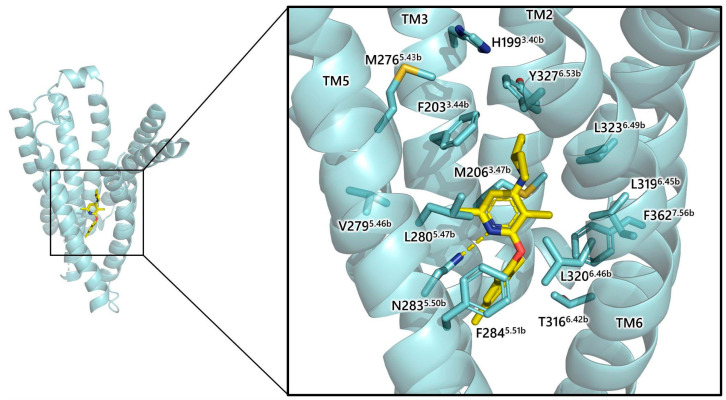
Allosteric binding of CP-376395 (C_21_H_30_N_2_O) to CRF_1_R (PDB: 4K5Y). Receptor residues are denoted by superscripts indicating their positions in receptor transmembrane domains (TMs). In subfamily B1 GPCRs, the most conserved residue in each TM is labeled as position index .50, preceded by the TM number (TM1-TM7). For example, Phe284^5.51b^ denotes Phe284 located in TM5, one residue after the most conserved residue, Asn283^5.50b^.

The J-domain of CRF receptors plays an important role in ligand binding and receptor activation. It contains contact sites of ligands, and is responsible for the transmission of conformational changes, associated with agonist binding and receptor activation, to the intracellular regions of receptor, which subsequently stimulate the G-proteins, thus resulting in a biological response [101,102]. Interaction of non-peptide antagonists, such as antalarmin and CP-376395 with TM residues of the J-domain of CRF_1_R allosterically inhibit agonist binding and block the receptor activation-associated conformational changes, thus antagonizing the CRF biological effects [1,102,103]. In detail, the pyrimidine nitrogen of CP-376395 interacts through a hydrogen bond with Asn283^5.50b^ of CRF_1_R, whereas the aryloxy group of ligand interacts with a hydrophobic pocket of receptor formed by Phe 284^5.51b^, Leu287^5.54b^, Ile290^5.57b^, Thr316^6.42b^, Leu319^6.45b^ and Leu320^6.46b^ [103] (Figure 6). 

The exocyclic alkylamino moiety of CP-376395 interacts with Gly324^6.50b^, Phe203^3.44b^, Leu280^5.47b^, Leu323^6.49b^, and Tyr327^6.53b^ of CRF_1_R [103]. The pattern of binding of the structurally related antalarmin is similar to that of CP-376395 [102]. The superscripts of receptor residues represent their positions in the TMs of the receptor, with the most conserved residue in each TM of subfamily B1 GPCRs to be assigned the position index .50, and this number is preceded by the TM number (TM1–TM7) [104]. For example, Phe284^5.51b^ denotes Phe284 located in TM5, one residue after the most conserved residue, Asn283^5.50b^.

In addition to the interaction with the non-peptide analogs, the residues of the J-domain interact with the CRF peptides. An alanine mutagenesis study has shown that Trp259^EL2^ and Phe260^EL2^ of the CRF_1_R play role in receptor interaction with CRF and SVG [101]. The superscripts EL1, EL2, and EL3 of receptor residues represent the ELs of the receptor, in which these residues are located. This interaction is supported by the cryoelectron microscopy (cryo-EM) results of CRF_1_R in complex with the G_s_ protein and CRF (receptor structure at 2.91 Å resolution, pdb: 6P9X) [105]. Specifically, the backbone of Trp259^EL2^ of the receptor is linked through a water-coordinated hydrogen bond network with Asn196^3.37b^, Tyr272^5.39b^, and Asp269^5.36b^, whereas the backbone of Phe260^EL2^ interacts through an H-bond with Arg^16P^. 

Similar to EL2, the EL1 and EL3 of CRF_1_R are also important for peptide binding. A photo-cross-linking study demonstrated that the amino-terminally located residues 17 and 22 of the UcnI analogs lay in close proximity to a region between Trp170^EL1^ to Glu179^EL1^ of CRF_1_R [106]. A different study has also shown that position 185 ^EL1^ of human CRF_2_R (189^EL1^ of human CRF_1_R) plays a role in receptor function. Specifically, SVG and UI bound with increased affinities to CRF_2_R when arginine was at position 185^EL1^ compared to the presence of histidine at this position [107]. In addition, the substitution of Tyr346^EL3^, Phe347^EL3,^ and Asn348^EL3^ of CRF_1_R by Ala significantly reduced the binding affinity of CRF [108]. Furthermore, a recent photocrosslinking study has revealed several crosslinking pairs between UcnI and CRF_1_R, namely the pairs Gln273^EL2^-Asp^8p^, Phe330^EL3^-Asp^8p^, Leu329^EL3^-His^12p^, Phe330^EL3^-His^12p^, Asn333^EL3^-His^12p^, Ile345^EL3^-His^12p^, Asn348^EL3^-His^12p^, Ser349^EL3^-His^12p^ and Ser349^EL3^-Leu^14p^ [109].

The pairs Gln273^EL2^-Asp^8p^, Asn348^EL3^-His^12p,^ and Ile345^EL3^-His^12p^ are also observed in the cryo-EM structure of CRF_1_R [100]. However, other pairs of crosslinking were not consistent with the Cryo-EM structure. Based on these results, the authors suggested that several regions of receptor containing crosslinked amino acids are subjected to large conformational changes during activation of the receptor, in contrast to the cryo-EM structure of receptor in complex with ligand and G-protein, which represents the most stable conformational active state [100].

In addition to the interaction of EL1 and EL2 with ligands, these regions contain two Cys, which are highly conserved among GPCRs, and form a disulfide bond that connects these loops, playing an important functional role [110,111]. Mutation of Cys188^EL1^ and/or the Cys258^EL2^ of CRF_1_R to different amino acids broke this disulfide bond and largely decreased the binding of CRF [97].

**Figure 7:**
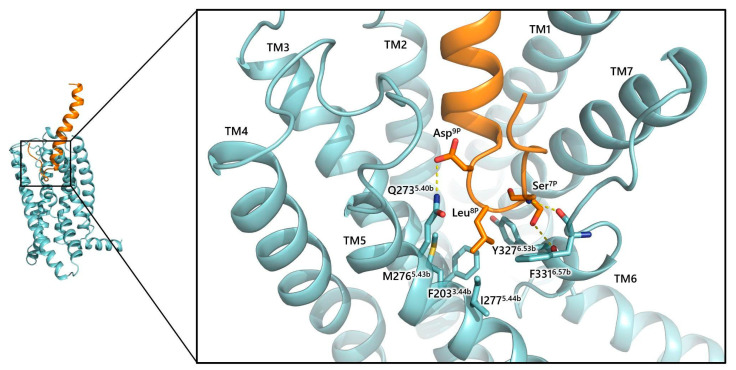
Molecular interactions of crucial CRF amino acids (orange) with CRF_1_R (light blue) (PDB: 6P9X). CRF forms an inserted loop consisting of residues Ser^7P^, Leu^8P,^ and Asp^9P^, to accommodate accordingly and form selective polar and hydrophobic interactions with amino acids of mainly TMs 3, 5, and 6. Polar interactions are depicted in yellow dashes.

The CRF also interacts with TM helices of CRF_1_R, excluding TM4 [105]. Specifically, the peptide enters the top of the receptor core, and forms interactions with the top of TM1 and TM2/EL1. As the peptide goes deeper into the receptor, it forms additional interactions with TM1 and EL2 at the outer membrane level and TM3, TM5, and TM7 deep into the pocket (Figure 7). The N-segment of peptide forms a loop in the receptor which orients toward the extracellular side of the receptor and forms polar interactions mainly with TM6, and secondarily with TM5 and EL2 residues. Residues at positions 7 to 9 of the N-segment of CRF have been characterized to play a crucial role in peptide’s agonist activity [112]. Specifically, Ser^7P^ forms an H-bond with the backbone of Phe331^6.57b^ and Tyr327^6.53b^ of the receptor (Figure 7). In addition, Leu^8P^ forms hydrophobic interactions in the hydrophobic region of the receptor consisting of Tyr327^6.53b^, Phe203^3.44b^, Ile277^5.44b,^ and Met276^5.43b^ and Asp^9P^ forms an H-bond interaction with Asn273^5.40b^, among others (Figure 7).

**Figure 8:**
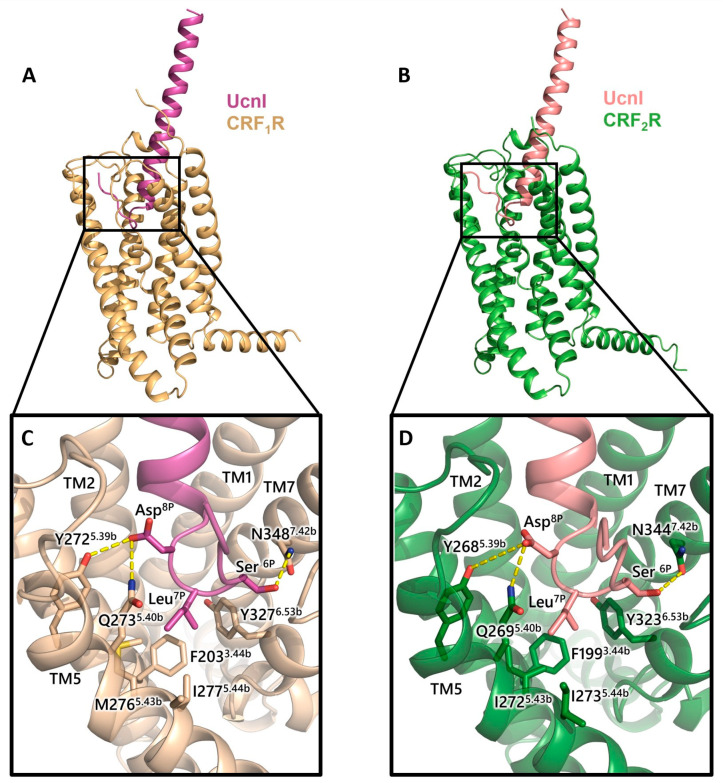
(**A**) Cryo-EM structure of UcnI in complex with TMD of CRF_1_R (PDB: 6PB0). (**B**) Cryo-EM structure of UcnI in complex with TMD of CRF_2_R (PDB: 6PB1). (**C**) Molecular interactions of crucial UcnI amino acids (magenta) with CRF_1_R intracellular residues (wheat). (**D**) Molecular interactions of UcnI amino acids (pink) with CRF_2_R (green). Polar interactions are shown in yellow dashes.

In addition to the structural information of CRF_1_R in complex with CRF, a recent Cryo-EM study has revealed the structure of both CRF_1_R and CRF_2_R obtained in complex with UcnI and G_s_ protein (Figure 8A and Figure 8B, respectively) [100]. UcnI interacts with the ELs and TM helices of CRF receptors, excluding TM4. Similarly, to the CRF peptide, UcnI enters the top of the receptor core, and forms interactions with the top of TM1 and TM2/EL1 of both receptors. The N-terminus of UcnI loops back up inside the receptors and forms interactions mainly with TM7. The amino acids 6 to 8 of the N-segment of UcnI have been shown to play a crucial role in the activity of the peptides. Specifically, Ser^6p^ of UcnI forms an H-bond with Asn348^7.42b^ of CRF_1_R (Figure 8C), which is also observed in the structure of CRF_2_R (Ser^6p^–344^7.42b^ interaction) (Figure 8D). Moreover, Asp^8p^ interacts with Tyr272^5.39b^ and Gln273^5.40b^ of CRF_1_R, similarly to its mode of interaction with Tyr268^5.39b^ and Gln269^5.40b^ in CRF_2_R (Figure 8C,D). An additional important feature of Asp8^p^, as revealed in the cryo-EM structure of CRF receptors, is its intramolecular electrostatic interaction with the Arg15^p^ of UcnI, thus stabilizing the bound peptide conformation [100]. In the structure of the CRF_1_R-UcnI complex, Ile^7p^ interacts with a hydrophobic region consisting of Tyr327^6.53b^, Phe203^3.44b^, Ile277^5.44b^, and Met276^5.43^, whereas the same hydrophobic region in CRF_2_R has a differentiation at position 276^5.43b^, where there is an isoleucine instead of a methionine (Figure 8C,D).

## 5. The Two-Step Model of Ligand–Receptor Interaction

The CRF peptides interact with the CRF receptors according to a two-step model. In the first step, the C-segment of the peptides interacts with the ECD of the receptors. This interaction orients the peptides in such a way that their N-segment residues interact with the J-domain of the receptors in the second step. This model was built based on the experimental results from the study of Hoare et al. [113]. Previous pharmacological studies have also shown the interaction between the ECD of CRF receptors, and the C-segment of the peptides [72,74,90,94]. In addition, other studies have shown the interaction between the amino acids of the N-segment of CRF peptides with the J-domain of CRF receptors [101,106,114,115]. The last interaction is responsible for receptor activation [116]. Specifically, Nielsen et al. have shown that replacement of the ECD of CRF_1_R with the first 16 N-segment residues of CRF constitutively activated the receptor because it mimicked the first-step interaction and allowed the important for receptor activation second-step interaction [116]. The constitutive activity of this chimeric receptor, which lacks the ECD, was not blocked by astressin which binds to the ECD of CRF_1_R [116]. In contrast, the small non-peptide allosteric antagonist, antalarmin, which binds to the TMs of CRF_1_R, decreased the constitutive activity of this construct. Interestingly, the Ala mutation of Leu^8p^ in the tethered N-segment of CRF abolished the constitutive activation of the chimeric receptor, suggesting the important role of this peptide residue in receptor–ligand interactions [116].

## 6. Structural Basis of Receptor Activation

The activation of CRF_1_R involves a complex and finely tuned set of structural interactions between different receptor residues, between peptide and receptor amino acids, and between receptor and G-protein residues. The high-resolution structures of CRF receptors provide a detailed view of these interactions. The receptor core, inclusive of all loops and the G_as_ domain, is resolved with great precision. This clarity allows for the accurate placement of side-chain rotamers and a deep understanding of the receptor’s structural conformation upon ligand binding.

Interactions of agonist peptides with the transmembrane helices (TM1–TM7) and extracellular regions of the receptor are characterized by a combination of hydrogen bonds, hydrophobic interactions, as described previously, and structural water molecules that stabilize the peptides within the receptor. The presence of structural water molecules in CRF receptor structures contributes significantly to the stability and specificity of peptide binding, by linking key residues such as E352^7.46b^ and Y195^3.36b^ that are essential for maintaining the receptors in an active conformation [105]. As revealed by the comparison of the inactive and active crystal structures of CRF receptors, their activation is associated with conformational changes, such as the reorganization of EL2, which includes an upward movement of TM4 and TM5 that repositions both EL2 and IL2 [105]. In addition, the computational data from a recent pharmacological study have proposed a movement of TM3 and TM5 of CRF_1_R during its activation [102]. Strengthening the interface between TM3 and TM5 by appropriately mutating their amino acids further stabilized the inactive state of the receptor [102].

**Figure 9:**
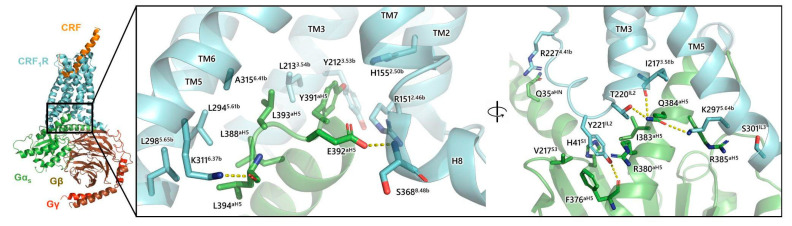
Molecular interactions of G_as_ protein (green) with CRF_1_R (cyan) (PDB: 6P9X). CRF (orange) and the three subunits of G-proteins, G_β_ protein (brown), and G_γ_ protein (red) are also shown as part of the general complex in the left side.

The CRF_1_R-G_as_ protein interface is another critical aspect of receptor activation. The receptor makes extensive contacts with G_as_, predominantly through hydrophobic interactions and hydrogen bonds across TM2, TM3, IL2, TM5, TM6, and the junction of TM7 and helix 8 [105]. Helix 8 is a structure of receptors located intracellularly after the end of TM7 and connects the TMs of the receptor with its C-domain. Specifically, Y391^aH5^ of G_as_ protein participates in hydrophobic interactions with H155^2.50b^, L213^3.54b^, Y212^3.53b^, and R151^2.46b^ of CRF_1_R, in addition to the H-bond networks in which participates as described in the study of Liang et al. (Figure 9) [105]. E392^aH5^ of G_as_ protein interacts through a hydrogen bond with the main chain of S368^8.48b^ located in helix 8, and L393^aH5^ of G_as_ protein participates in hydrophobic interactions with A315^6.41b^ and L294^5.61b^ of the receptor (Figure 9). In addition, the main chain oxygen of L394^aH5^ of G_as_ protein interacts with receptor residues, K311^6.37b^ forming a hydrogen bond and L298^5.65b^ by hydrophobic interactions. Residues from the alpha helix 5 of the G_as_ protein, and specifically, Q384^aH5^ make H-bond interactions with K297^5.64b^, K334^5.64b^, T258^IL2^ and L255^3.58b^. In addition, T220^IL2^ interacts with main chain atoms of I217^3.58b^, while I383^aH5^ and R380^aH5^ participate in hydrophobic interactions with T220^IL2^ and Y221^IL2^. Y221^IL2^ also forms a hydrogen bond with the main chain oxygen of F376^aH5^ in the G_as_ protein, whereas Q35^aHN^ interacts with R227^4.41b^. Moreover, R385^aH5^ participates in hydrophobic interactions with K297^5.64b^ (Figure 9). The superscripts IL1, IL2, and IL3 of receptor residues represent the intracellular loops (ILs) of the receptor, in which these residues are located. The superscript aH5 represents the alpha helix 5 of the G_as_ protein. Similar to CRF_1_R, the α5 helix of the G_αs_ protein extensively interacts with TM2, TM3, TM5, TM6, IL2, and IL3, and the TM7-H8 junction of the CRF_2_R, highlighting the extensive interface for G-protein coupling [117]. Specifically, Y391 in the G_as_ protein binds to a sub-pocket formed by R148^2.46b^, H152^2.50b^, and E205^3.50b^, Y208^3.53b^, L209^3.54b^ of CRF_2_R. Other interface residues in the α5 helix of the Gαs protein include E392, forming polar contacts with K310^6.40b^ and N363^8.47b^ of the receptor, and Q390 forming a hydrogen bond with R148^2.46b^ of the receptor. In addition, the C-terminal L394 of the G_as_ protein forms a charge interaction with K307^6.37b^ of the TM6 of the receptor. 

The IL2 of CRF receptors plays an important role in their interaction with different G-proteins, consistent with the ability of subfamily B1 GPCRs to engage with multiple G-proteins and their related signaling pathways. Conformational changes in IL2 of CRF receptors are key in differentiating their interactions with various G-proteins (G_s_, G_11_, and G_o_). Specifically, the α5 helices of different G-proteins interact differently with the receptor [117]. The sharp kink in the middle of TM6 of CRF_2_R facilitates the formation of an open G-protein-binding pocket, allowing for the accommodation of the relatively large C-termini of the α5 helix of Gα subunits, particularly G_s_ [117].

The structural–functional knowledge derived from CRF receptors can provide valuable insights into the activation mechanisms of subfamily B1 GPCRs. Common structural features of CRF receptors and subfamily B1 GPCRs have been extensively studied. The formation of a cytoplasmic cavity by three intracellular loops (ILs) for G-protein coupling, the interaction of the α5 helix of G_αs_ proteins with TM2, TM3, and IL2, which is a highly conserved feature across subfamily B1 GPCRs, suggest a common mechanism in G_s_ protein coupling for these receptors. The ECD and ELs of subfamily B1 GPCRs, including CRF receptors, show the ability to regulate the binding of different peptides, indicating that the general principles of ligand recognition and binding are shared across this subfamily. Similar to CRF receptors, subfamily B1 GPCRs undergo conformational changes upon activation, such as the outward movement of TM6/ECL3/TM7, which are variable among different receptors but follow a common trend [57].

However, there are structural aspects of activation within the CRF receptor family which are not necessarily extrapolatable. These are the specific interactions at the residue level, and the diverse G-protein engagement. Differences between receptors of this family are likely reflective of the distinct dynamics of side chains and backbone conformations within individual receptors. Thus, although CRF receptors provide a valuable framework for understanding subfamily B1 GPCR activation, variations in ligand recognition, G-protein engagement, and structural dynamics of these receptors highlight the importance of studying each of them individually. 

## 7. Molecular Mechanisms of Ligand Selectivity

### 7.1. Selectivity of Non-Peptide Antagonists

The crystal structure of the inactive state of CRF_1_R has revealed that a layer formed by the side chains of several residues, including His199^3.40b^ and Met276^5.43b^, is located just above the bound CP-376395 [103]. Interestingly, although His199^3.40b^ and Met276^5.43b^ do not interact with the ligand, their mutation to the corresponding residues of CRF_2_R (V195^3.40b^ and I272^5.43b^, respectively) largely reduces non-peptide antagonist binding to CRF_1_R [118]. The non-conserved His199^3.40^ and Met276^5.43^ interact with the conserved Tyr327^6.53b^ and Phe203^3.44b^, possibly affecting the positioning of these two aromatic residues. It has been proposed that during ligand binding and dissociation Tyr327^6.53b^ and Phe203^3.44b^ change rotameric states, such as to allow the entrance and exit of the small non-peptide ligands from the CRF_1_R [98]. In contrast, the mutation of His199^3.40b^ and Met276^5.43b^ of CRF_1_R to alanine did not affect non-peptide ligand binding, since this small well-tolerated amino acid does not largely affect the conformation of receptor [102,119]. These conformational changes provide a theoretical rationale for the CRF_1_R-selectivity of non-peptide ligands given that all receptor residues directly interacting with these molecules are completely conserved in CRF_2_R. It is possible that in CRF_2_R, the corresponding Val195^3.40b^ and Ile272^5.43b^ restrict the aromatic residues, which correspond to Tyr327^6.53b^ and Phe203^3.44b^ of CRF_1_R, to a conformation that prohibits the access of non-peptide ligands to their binding sites [98].

### 7.2. Selectivity of Peptide Agonists

Residues at the N-segment of the CRF and related peptides play an important role in CRF receptor selectivity. Specifically, the replacement of the motif Thr^11p^-Phe^12p^-His^13p^ of CRF with the corresponding motifs of UcnII and UcnIII, Pro^11P^-Ile^12P^-Gly^13P^ and Pro^11P^-Thr^12P^-Asn^13P^, respectively, conferred to peptide CRF_2_R-selectivity [120]. Supportive evidence for the structural importance of the Thr^11p^-Phe^12p^-His^13p^ motif of CRF in receptor selectivity is provided by the study of Isfort et al. [121]. Specifically, Phe, Leu, Ile, Thr, Gln, His and Tyr at position 12 and Phe, Gln, Trp, Tyr, Val, Ile, Leu and 2-naphthylalanine at position 13 are the preferable substitutions for CRF_2_R selectivity. In addition to the N-region, amino acids at the C-region of the CRF and related peptides are significant for CRF receptor selectivity. Importantly, Arg^35p^ and the acidic residue (Asp or Glu) at position 39 are not conserved in the CRF_2_R-selective UcnII and Ucn III, which have an Ala at these positions, suggesting that they could play an important role in the selective binding of these peptides to CRF_2_R. Simultaneous replacement of Arg^34p^ (Arg^35p^ of CRF) and Asp^38p^ (Glu^39p^ of CRF) of SVG by the corresponding Ala^35p^ and Ala^39p^ of UcnII increased the CRF_2_R-selectivity obtained after the substitution of Ser^10p^ of SVG (which corresponds to Thr^11p^ of CRF) by the corresponding Pro^11p^ of UcnII and UcnIII, [122]. Similarly, the corresponding substitutions in the UcnI increased CRF_2_R-selectivity, whereas in the CRF it resulted in a decrease in binding to both receptor subtypes (EC_50_ > 100 nM for CRF_2_R and EC_50_ > 1000 nM for CRF_1_R), suggesting that UcnI, SVG, and CRF bind to CRF receptor with slightly different modes, which might be differentially affected by the same modification of peptides [122]. 

The molecular determinants of peptide selectivity could be determined by examining the existing structures of CRF_1_R and CRF_2_R and comparing the amino acids at positions 11-13 of the N-segment of CRF (Thr^11P^-Phe^12P^-His^13P^), UcnI (Thr^11P^-Phe^12P^-His^13P^), UcnII (Pro^11P^-Ile^12P^-Gly^13P^) and UcnIII (Pro^11P^-Thr^12P^-Asn^13P^) bound to the two CRF receptor types. Given that Pro^11P^ is an alpha-helix breaker, these residues abate the α-helicity, leading to an impairment of binding to CRF_1_R [120]. Moreover, a comparison of the electrostatic surface potentials of the ECD for both CRF receptors indicates that Arg^35P^ (positively charged amino acid) present in CRF and UcnI is compatible for interaction with Glu104 of CRF_1_R as well as the corresponding Pro100 in CRF_2_R. In contrast, UcnII and UcnIII have Ala^35P^, the hydrophobic surface of which may interact only with Pro100 of CRF_2_R and not with Glu104 of CRF_1_R, which could provide an additional determinant of peptide selectivity [123].

Receptor selectivity could also be attributed to modifications of different sets of amino acids at positions 30, 31, 33, 34, and 35 and/or to a change in the conformation of peptide by introducing lactam bridges at different positions of CRF [124,125]. For example, Rivier et al. have shown that the introduction of a Glu^32p^-Lys^35p^ lactam bridge into a CRF analog with the eleven N-terminal residues deleted (creating the analog, cyclo(32-35)[DPhe^12^,Nle^21,38^,Glu^32^,Lys^35^]- hCRF(12-41)) yielded a CRF_2_R-selective ligand [124]. In marked contrast, the introduction of a Glu^30p^-Lys^33p^ lactam bridge in a CRF truncated analog created the non-selective astressin ([cyclo(30-33)[DPhe(12),Nle(21),Glu(30), Lys(33),Nle(38)]hCRF((12-41))]) [125]. Furthermore, salt bridges that are formed in the Glu^31p^–Glu^34p^ region of CRF analogs could play a crucial role in peptide selectivity. The CRF analog Stressin1-A is a CRF_1_R-selective peptide with quite similar sequence as CRF (cyclo(31-34)[DPhe^12^,Nle^21,38^,Glu^31^,Lys^34^]Ac-hCRF_(4-41)_). In Stressin1-A, residues Glu^31p^ and Lys^34p^ form a lactam bridge resulting in a 130-fold selectivity increase towards the CRF_1_R [126]. Interestingly, the linear counterpart of stresin-1A, (linear[DPhe^12^, Nle^21,38^,Glu^31^,Lys^34^]-Ac-hCRF(4-41)), which has Glu and Lys at positions 31 and 34, respectively, displayed CRF_1_R selectivity, similar to stressin-1 A, whereas the linear analog [DPhe^12^,Nle^21,38^,Glu^30^,Lys^33^]-Ac-hCRF(4-41), which has Glu and Lys at positions 30 and 33, respectively, was non-selective [126].

Differential truncation of peptides could also confer the selective binding of peptides to the two CRF receptor types. Thus, although the introduction of a cyclic constraint between Glu^32p^ and Lys^35p^ of a truncated CRF analog created the CRF_2_R-selective ligand (cyclo(32-35)[DPhe^12^, Nle^21,38^,Glu^32^, Lys^35^]- hCRF(12-41)), the same modification in a lengthier CRF analog created the non-selective ligand cyclo(32-35)[DPhe^12^,Nle^21,38^, Glu^32^,Lys ^35^]-Ac-hCRF(4-41) [124,126].

## 8. Concluding Remarks

In this exploration of CRF peptides and their receptors, we have delved into the intricate structural and functional nuances that govern their roles in diverse physiologic and pathologic states. The elucidation of the crystal structures of CRF receptors, particularly CRF_1_R and CRF_2_R, has been pivotal in enhancing our understanding of their activation mechanisms and ligand specificity.

The detailed insights into the interaction dynamics between CRF peptides and their receptors underscore the sophistication of ligand–receptor binding, ligand selectivity for the CRF_1_R and CRF_2_R, and activation processes. In addition, these interactions, characterized by specific amino acid interactions, and conformational changes, highlight the intricacy of G-protein-coupled-receptor (GPCR) signaling. 

Moreover, the structural–functional insights gained from CRF receptors provide a valuable framework for understanding the function of the broader subfamily B1 GPCRs. While certain aspects of CRF receptor activation and ligand binding can be generalized to other receptors in this subfamily, the unique features of each receptor must be appreciated. This knowledge underscores the potential for developing targeted therapies that harness the specific characteristics of each receptor within this subfamily.

The revelation of the molecular mechanisms underlying receptor activation and ligand selectivity has profound implications for therapeutic applications. Understanding the structural basis of receptor activation opens avenues for the development of novel drugs targeting CRF receptors. These drugs hold significant promise in the treatment of a range of conditions, from psychiatric disorders such as depression and anxiety, to those of the cardiovascular, gastrointestinal, and immune systems. As we continue to deepen our understanding of these molecular mechanisms, we edge closer to unlocking the full therapeutic potential of targeting CRF receptors and their related signaling pathways.

## Figures and Tables

**Table 1:** Amino acid sequences of peptides of the CRF family, their percentage of identity to h/rCRF, and their binding affinities to two CRF receptor types. Highly conserved residues are in bold. K_i_ is the binding affinity of peptides mostly determined from heterologous displacement radioligand experiments.

Peptide		Amino Acid Sequence	K_i_ (nM)		% h/rCRF Identity	
			**CRF_1_R CRF_2_R**			
h/rCRF ^1^	Agonist	SEEPPI**SLD**LTFH**LLR**EV**LE**MARAEQLAQQ**A**HS**N**RK**LM**EI**I**	1.9 ^b^	31 ^a^	100	
oCRF	Agonist-CRF_1_R	SQEPPI**SLD**LTFH**LLR**EV**LE**MTKADQLAQQ**A**HS**N**RK**LL**DIA	1.2 ^b^ 1.1.2 ^b^	185 ^a^	82.9	
UI	Agonist	NDDPPI**SID**LTFH**LLR**NM**IE**MARIENEREQ**A**GL**N**RK**YL**DE**V**	0.4 ^b^	2.2 ^a^	53.7	
SVG	Agonist	ZGPPI**SID**LSLE**LLR**KM**IE**IEKQEKEKQQ**A**AN**N**RL**LL**DT**I**	0.7 ^b^	4.3 ^a^	45.0	
α-helCRF ^2^	Antagonist	**D**LTFH**LLR**EM**LE**MAKAEQEAEQ**A**AL**N**RL**LL**EEA	23.7 ^b^	96 ^a^	67.9	
Astressin	Antagonist	H**LLR**EV**LE**BARAEQLAQE**A**HK**N**RK**L**BEI**I**	15.4 ^b^	1.5 ^b^	86.2	
rUcnI ^1^	Agonist	DDPPL**SID**LTFH**LLR**TL**LE**LARTQSQRER**A**EQ**N**RI**I**FDS**V**	0.3 ^b^	0.3 ^b^	45.0	
hUcnI ^1^	Agonist	DNPSL**SID**LTFH**LLR**TL**LE**LARTQSQRER**A**EQ**N**RI**I**FDS**V**	0.4 ^b^	0.3 ^b^	42.5	
hUcnII ^1^	Agonist-CRF_2_R	IVL**SLD**VPIG**LL**QIL**LE**QARARAAREQ**A**TT**N**AR**IL**AR**V**	>100 ^c^	1.7 ^c^	34.2	
mUcnII ^1^	Agonist-CRF_2_R	VIL**SLD**VPIGLL**R**IL**LE**QARYKAARNQ**A**AT**N**AQ**IL**AH**V**	>100 ^c^	2.1 ^c^	34.2	
hUcnII I ^1^	Agonist-CRF_2_R	FTL**SLD**VPTNIMNLLFNIAKAKNLRAQ**A**AA**N**AH**L**MAQ**I**	>100 ^c^	22 ^c^	31.6	
mUcnIII ^1^	Agonist-CRF_2_R	FTL**SLD**VPTNIMNILFNIDKAKNLRAK**A**AA**N**AQ**L**MAQ**I**	>100 ^c^	5 ^c^	26.3	

^1^ The abbreviations h/r, r, h, and m before the peptides refer to human/rat (see main text), rat, human and mouse, respectively; ^2^ α-helCRF is the α-helical CRF (9–41); ^a^ Grigoriadis et al., 1996 [60], ^b^ Dautzenberg et al., 2001 [61], ^c^ Lewis et al., 2001 [7].

**Table 2:** Amino acid sequences of chimeric peptides, created by linking the N-segment (DDPPLSIDLTFHLLRTLDEI) and the C-segment (QNRKLLDEV) of UcnI with charged linkers (in bold) of various lengths. The EC_50_ are the biopotencies of peptides (Adopted and modified by the study of Beyermann et al., 2000 [77]).

Ligand	EC_50_ (nM)
**Chimeric Peptides**	
N-region-**E K E E K E K K R K E**-C-region	0.6
N-region-**E K E K E K K R K E**-C-region	25
N-region-**E K K E K K R K E**-C-region	8.5
N-region-**E K E K K R K E**-C-region	0.9
N-region-**E K K K R K E**-C-region	12
N-region-**E K K R K E**-C-region	50
N-region-**E K R K E**-C-region	30
N-region-**E K K E**-C-region	4.6

## Data Availability

Data are contained within the article.
